# Microstructural Characterization and Mechanical Properties of L-PBF Processed 316 L at Cryogenic Temperature

**DOI:** 10.3390/ma14195856

**Published:** 2021-10-06

**Authors:** Pragya Mishra, Pia Åkerfeldt, Farnoosh Forouzan, Fredrik Svahn, Yuan Zhong, Zhijian James Shen, Marta-Lena Antti

**Affiliations:** 1 Materials Science, Department of Engineering Sciences and Mathematics, Luleå University of Technology, 97187 Luleå, Sweden; pia.akerfeldt@ltu.se (P.Å.); farnoosh.forouzan@ltu.se (F.F.); marta-lena.antti@ltu.se (M.-L.A.); 2 GKN Aerospace, 46130 Trollhättan, Sweden; fredrik.svahn@gknaerospace.com; 3 Department of Materials and Environmental Chemistry, Stockholm University, 10691 Stockholm, Sweden; zhongy5233@gmail.com

**Keywords:** 316 L stainless steel, cryogenic temperature, martensite, strain-induced martensite, L-PBF process

## Abstract

Laser powder bed fusion (L-PBF) has attracted great interest in the aerospace and medical sectors because it can produce complex and lightweight parts with high accuracy. Austenitic stainless steel alloy 316 L is widely used in many applications due to its good mechanical properties and high corrosion resistance over a wide temperature range. In this study, L-PBF-processed 316 L was investigated for its suitability in aerospace applications at cryogenic service temperatures and the behavior at cryogenic temperature was compared with room temperature to understand the properties and microstructural changes within this temperature range. Tensile tests were performed at room temperature and at −196 °C to study the mechanical performance and phase changes. The microstructure and fracture surfaces were characterized using scanning electron microscopy, and the phases were analyzed by X-ray diffraction. The results showed a significant increase in the strength of 316 L at −196 °C, while its ductility remained at an acceptable level. The results indicated the formation of ε and α martensite during cryogenic testing, which explained the increase in strength. Nanoindentation revealed different hardness values, indicating the different mechanical properties of austenite (γ), strained austenite, body-centered cubic martensite (α), and hexagonal close-packed martensite (ε) formed during the tensile tests due to mechanical deformation.

## 1. Introduction

Austenitic stainless steel 316 L is a widely used alloy within the aerospace and medical industries as well as in nuclear power plants due to its good mechanical properties at room temperature (RT) and cryogenic temperatures [1,2]. Recently, laser powder bed fusion (L-PBF) manufacturing of 316 L stainless steel has received much attention due to its improvement in mechanical properties compared to conventional manufacturing [3,4]. One drawback of conventional coarse-grained 316 L is its low yield strength (~250–300 MPa) [5] and several methods are used to strengthen the alloy, such as cold rolling and forging. However, this normally reduces the tensile ductility and strategies to achieve high strength and ductility remain rare despite decades of studies [6].

The L-PBF technique can produce 316 L with an extraordinary combination of strength and ductility [6,7]. In addition to improved mechanical properties, L-PBF offers the possibility to manufacture parts with complex geometries [8,9], and it reduces material waste as well as the buy-to-fly ratio, making it attractive for the aerospace industry [10]. In the L-PBF process, metal powder is melted layer-by-layer to form a near-net-shape component and due to the high cooling rate a unique microstructure is formed. It consists of a cellular structure with subgrains that are much finer than the grains formed by conventional manufacturing, as described by, for example, Liverani et al. [11]. Thus, L-PBF permits an opportunity to tailor the microstructure and subsequently the mechanical properties. Besides the high cooling rate, there are other factors contributing to the formation of the unique microstructure in L-PBF processes, a microstructure that is not obtainable through conventional methods. These factors include highly localized melting, a strong temperature gradient, and rapid solidification [6,12,13].

To the authors’ knowledge, there are few studies of the cryogenic properties of L-PBF-processed 316 L stainless steel. Bidulskỳ et al. [14] reported increased tensile strength and elongation at two cryogenic temperatures. The tensile strength was high, with a value of 1246 MPa at 4.2 K, and the elongation reached 55% at 77 K. For conventionally produced 316 L, there are more studies at cryogenic temperatures [15,16,17]. Tensile strengths of 1196 and 1328 MPa, yield strengths of 590 and 494 MPa, and elongations of 33% and 38%, respectively, at −160 °C of conventionally manufactured 316 have been reported by Lee et al. [18].

The microstructure strongly influences the mechanical properties (e.g., grain size, impurities, texture, residual stresses, and voids) [19]. At low temperatures, austenite may transform into martensite upon plastic deformation, i.e., strain-induced martensitic transformation [1,18,20,21,22,23,24]. In general, for conventionally manufactured 316 L, two sequences of phase change occur in the austenite to martensite transformation: γ to ε and then to α or γ to α. This transformation depends on several factors, such as twin formation, dislocation slip, and stacking fault energy [25,26,27]. The martensitic transformation makes the initially homogeneous microstructure become strongly heterogeneous with martensite platelets embedded in the austenitic matrix. Since BCC α-martensite is significantly more rigid than FCC γ-austenite, its presence influences the plastic flow and hardening [28,29].

The aim of the current paper is to investigate the suitability of using L-PBF 316 L for low-temperature applications. This is done through mechanical testing at −196 °C followed by microstructural characterization and fractography, X-ray diffraction (XRD), and nanoindentation hardness analyses.

## 2. Materials and Methods

### 2.1. Materials

The 316 L stainless steel bars with dimensions of 10 by 10 mm and 130 mm in length were built in the horizontal position in argon atmosphere using an EOSINT M270. The chemical compositions of the initial 316 L powder and of the L-PBF-processed bars are summarized in Table 1. The chemical composition of the powder was obtained from the supplier and the chemical composition of the L-PBF-processed bars was evaluated by spark optical emission spectroscopy (Spark-OES, LKAB, Luleå, Sweden). The processing parameters for L-PBF are described in Table 2. Five bars were selected for this study.

### 2.2. Experimental Methods

**Figure 1:**
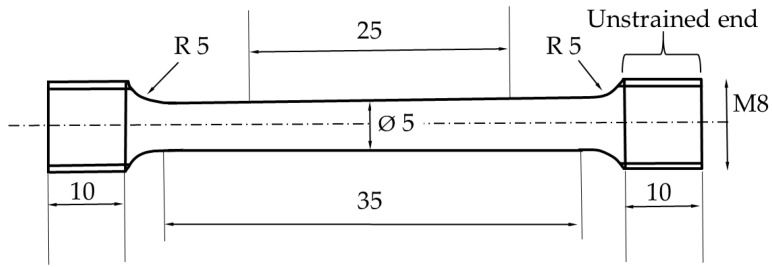
Drawing of the specimen design according to ISO 6892-1. Dimensions in mm.

From the five bars selected for this study, four specimens were machined for tensile testing according to standard ISO 6892-1 [30], see Figure 1, and one bar was maintained in the as-built condition. The smallest diameter for this standard was 5 mm, and the threads at the ends of each specimen were type M8. Tensile testing was performed at room temperature on specimens 1 and 2 and at −196 °C on specimens 3 and 4. Room temperature tensile tests were performed in a Servo-Hydraulic Instron 1272 instrument with a load cell of 20 kN. Tensile tests performed at −196 °C were conducted at Sandvik AB in accordance with standard SS-EN ISO 6892-3 [31], where the tensile test bars were cooled to −196 °C by immersing them in liquid nitrogen and keeping them there throughout the test. The sample was immersed in the nitrogen for 20 min before the start of the tests to ensure an even temperature profile and correct temperature (−196 °C). The tests were performed under strain control at a strain rate of 0.00025 s^−1^, and Rp0.2 was used for the yield strength.

For microstructural characterization, the specimens were prepared by conventional methods. First, the specimens were cut just below the fracture surface with a Struers Secotom–10, and the fracture surface was kept for fractography. The specimens were prepared without mounting, and the following steps were performed: (i) manual planar grinding, (ii) electropolishing in Struers LectroPol-5 mixed with A2 standard electrolyte, and (iii) electrolytic etching with oxalic acid (10 g of oxalic acid mixed with 100 mL of distilled water). Microstructural analysis was performed with a Nikon SMZ1270 optical light microscope and a scanning electron microscopy (JEOL JSM-IT 300 and Magellan 400 (Luleå University of Technology (LTU), Luleå, Sweden)). Fractography was performed on all tensile tested specimens with a JEOL JSM-IT 300 scanning electron microscope. The average grain size was measured from optical microscopy images using ImageJ. First, a random straight line is drawn through the micrograph and the number of grain boundaries intersecting the line are counted. Then, the average grain size is calculated by dividing the number of intersections by the actual line length. Average grain size = number of intersections/actual length of the line.

Phase compositions were analyzed by a PANalytical Empyrean X-ray diffractometer equipped (Luleå University of Technology (LTU), Luleå, Sweden) with a CuKα LFF HR X-ray tube. XRD patterns were obtained at room temperature over a 2θ range of 40° to 100°. The data were analyzed by High Score Plus software version 4.9 using the PDF 4 + (2021) database, and Rietveld refinement was performed to estimate the amount of different crystalline phases. Nanoindentation tests were performed on all tensile tested specimens and the as-built specimen (specimen 5). The nanoindentation tests were conducted using a NanoTest Vantage high-temperature nanoindenter (LTU, Luleå, Sweden). The maximum load applied on the surface was 60 mN, and a 10 × 10 indentation matrix was investigated. Vickers microhardness measurements were performed in a Matsuzawa MXT microhardness tester (LTU, Luleå, Sweden). A load of 100 g was used and in total 10 indentations were carried out on each investigated specimen.

## 3. Results and Discussion

### 3.1. Mechanical Properties

**Figure 2:**
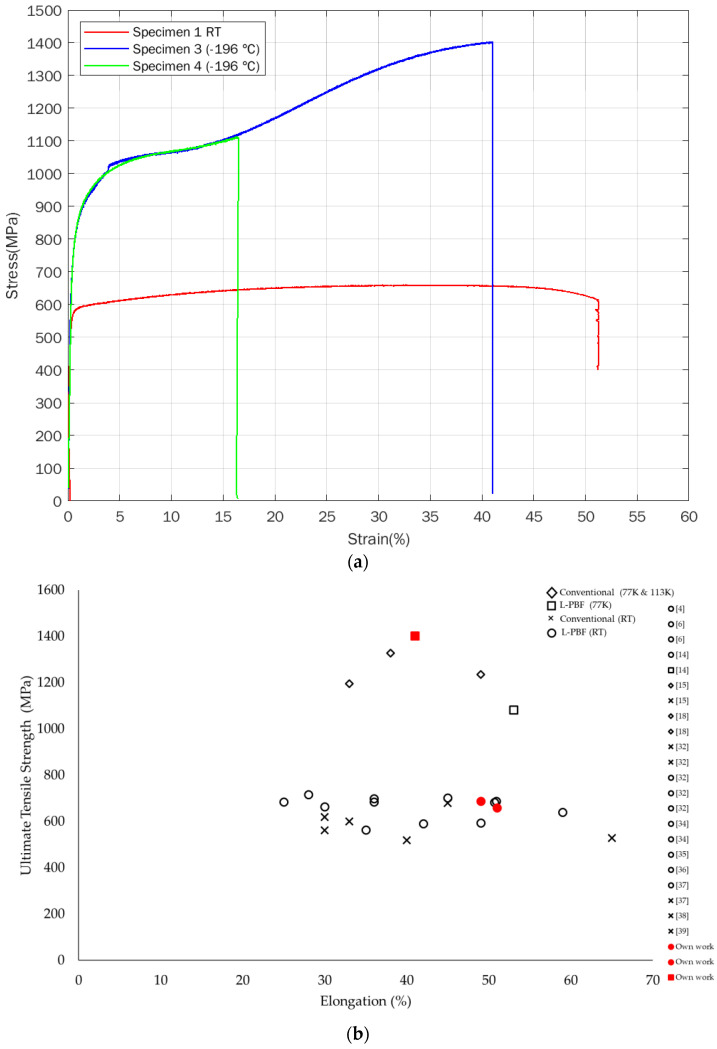
(**a**) Stress–strain curves for L-PBF-processed 316 L stainless steel at room temperature (RT) specimen 1 and −196 °C temperature specimens 3 and 4. (**b**) Comparison graph of ultimate tensile strength versus elongation for various 316 alloys conventionally built and L-PBF processed, at room and cryogenic temperatures, including own work.

The results of the tensile tests of L-PBF-processed 316 L performed at room temperature and at −196 °C are shown in Figure 2a and Table 3. At room temperature, the ultimate tensile strengths are 660 and 689 MPa, the yield strengths are 570 and 594 MPa, and the elongation values are 51 and 49%. At −196 °C, there is a significant increase in yield strength and particularly in ultimate tensile strength, with values of 751/770 and 1403/1113 MPa, respectively. The elongation at −196 °C is less than that at room temperature. One of the −196 °C temperature specimens (specimen 3) elongates by 41%, and the other specimen (specimen 4) elongates by only 16%. It should be noted that both the −196 °C temperature tensile specimens broke outside of the gauge length section, which means that these elongation values are lower than the actual elongation values in the ideal situation. As the specimens 3 and 4 were tensile tested at an identical temperature (−196 °C), with all other test conditions the same, the results of the tensile tests should be in the same range. Differences, like the ones reported in this work, could be caused by other reasons, such as sample preparation, etc. [19]. However, due to the strain-induced martensite, there is a high sensitivity to crack initiation and growth and hence unexpected fractures are common [15,28].

It has been shown that L-PBF-manufactured 316 L generally has improved mechanical properties compared to conventionally manufactured 316 L [32,33,34]. Figure 2b and Table 4 show comparisons between various 316 alloys manufactured by conventional methods and with the L-PBF process, tested at room and cryogenic temperatures, including data from our work. It is clearly seen that the strength at cryogenic temperatures is significantly higher than at room temperature. The superior strength is believed to be due to a higher concentration of dislocations and twins formed due to plastic deformation during the tensile test [32,33,34].

It can be seen from Figure 2a that the room temperature curve (specimen 1) shows typical strain hardening and ductility behavior. The −196 °C curves, corresponding to cryogenic specimens 3 and 4, exhibit different behavior compared to the room temperature specimen. The strain hardening increases with increasing strain up to 16%. At this stage, a second hardening starts. Specimen 4 breaks at 16% strain, but for specimen 3, the second hardening continues, and the specimen continues to be strained up to 41% strain at fracture. Martensite formation resulting from plastic deformation of austenite (γ) is of great interest for producing high strength and ductility in austenitic stainless steels. At the beginning of the deformation, the austenitic γ phase is identified when the martensite content is negligible and does not affect the strain hardening process. The kinetic increase in strain hardening corresponds to the restricted mobility of dislocations in the γ phase due to the presence of α martensite sites that create local stress fields associated with the minimization of atom movement during the change of the crystal structure [18,21,40].

The second hardening at −196 °C has been reported by others [16,17,40], who discussed the tensile deformation behavior of SS 316 L at room temperature and cryogenic temperature. The authors observed multiple strain hardening stages at cryogenic temperatures. Strain hardening increases with increasing strain due to plastic deformation. Similar behavior has been reported for 304 and 316 stainless steel by Kyung Jun Lee et al. [18] and Li et al. [41]. They reported a significant increase in strength at cryogenic temperature during the second hardening stage, which is believed to be due to phase transformation-induced strain hardening. The austenite to martensite transformation is minimal at room temperature, but that the martensite transformation could explain the enhanced strength and reduced ductility of 316 L stainless steel at cryogenic temperatures [40,42].

### 3.2. Microstructures

**Figure 3:**
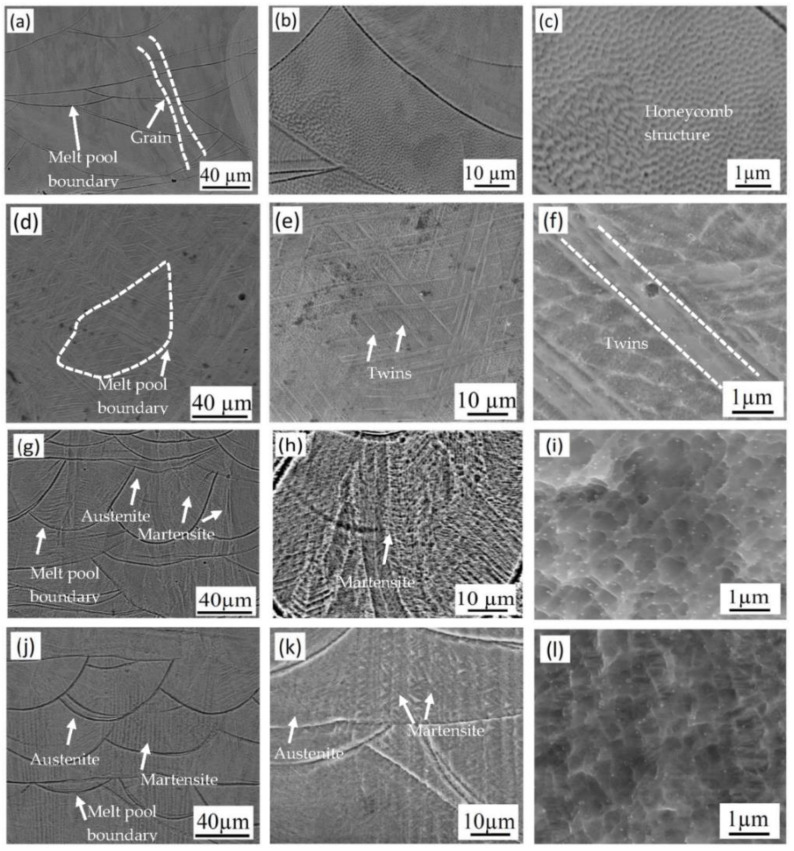
SEM images of 316 L stainless steel: (**a**–**c**) as-built specimen 5, (**d**–**f**) room temperature specimen 1, (**g**–**i**) −196 °C specimen 3, and (**j**–**l**) −196 °C specimen 4 after electrolytic etching with oxalic acid.

SEM images of the as-built specimen and the specimens tested at room temperature and at −196 °C are shown in Figure 3. The microstructure of the as-built specimen (Figure 3a–c) is typical for L-PBF-processed 316 L, in which layer-by-layer overlapping of melt tracks results in variable melt pool boundaries between tracks. Elongated grains are also observed, crossing the melt pool boundaries, which is also characteristic for additively manufactured material because of the thermal gradient in the material during the building process. A subgrain structure can be observed in the melt pool, as shown in Figure 3b. At higher magnification, see Figure 3c, a fine cellular, honeycomb structure was observed. This unique microstructure of L-PBF 316 L has been observed earlier [4,35,43].

Several twins are observed after tensile deformation, as shown in Figure 3d–f. This is common for deformed austenitic structures; it has been confirmed for L-PBF-processed 316 L [19,33,34]. It is worth mentioning that in the present study, no martensite was identified during microstructure characterization of room temperature samples [33,44,45].

The microstructures of cryogenic specimens 3 and 4 are shown in Figure 3g–l. Compared to the room temperature specimens, different microconstituents are observed. The presence of a thin and parallel plate-like structure in specimens 3 and 4 suggests that martensite is formed during deformation at −196 °C.

Compared to conventionally manufactured, L-PBF-manufactured 316 L has a unique microstructure with fusion boundaries and a very fine subgrain structure. As a result, the L-PBF 316 L shows both high strength and elongation [6,14,19,24]. The very fine grain size could also play a vital role in the mechanical properties at −196 °C [14,25,46]. According to the Hall–Petch relationship, a reduction in grain size contributes to the significantly high yield and tensile strength. In addition, at cryogenic temperatures during deformation, it is suggested that the strain-induced martensite transformation also contributes to an improved strength [14]. This is further supported in the literature when thin, parallel, and needle-like structures are observed in austenite grains after plastic deformation [1,20].

According to the Nohara et al. [47] equation, the Md (30/50) value of the specimens in the current study, with the chemical composition specified in Table 1, is −47.8 °C (the temperature at which 50% martensite forms at 30% true strain) when the average grain size is approximately 50 µm (5.5 ASTM). However, measuring the subgrain sizes of L-PBF 316 L is challenging since the grains, besides being very small, are twisted and bent [48,49]. In the literature, others have reported the average grain size of L-PBF 316 L to be 10–70 µm [33,49,50]. Therefore, one should keep in mind that the Md temperature varies, and in this case for the very fine microstructure, it could be hard to estimate in a satisfactory way.

### 3.3. Fractography

**Figure 4:**
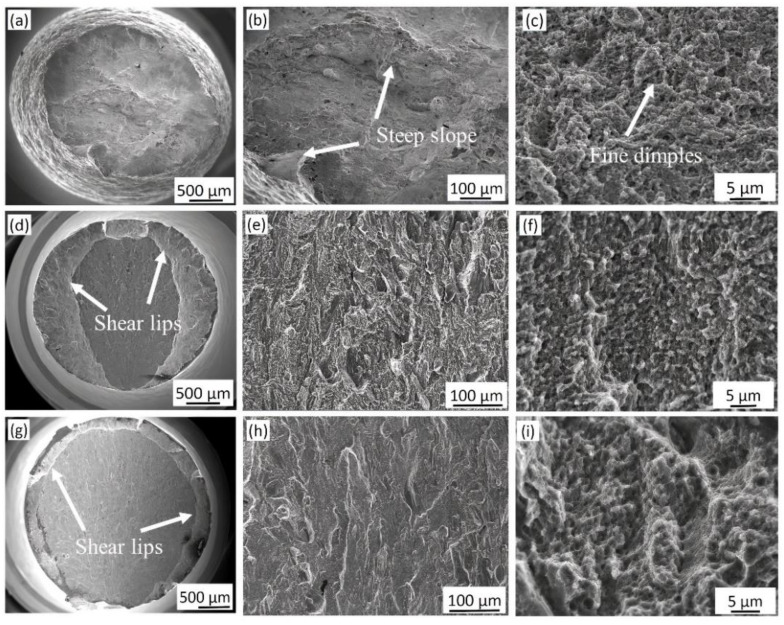
SEM images of the fracture surfaces and dimples of 316 L: (**a**–**c**) room temperature specimen 1, (**d**–**f**) −196 °C specimen 3, and (**g**–**i**) −196 °C specimen 4.

**Figure 5:**
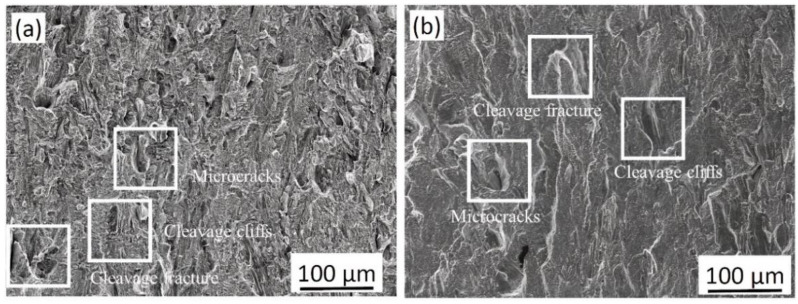
The fracture surface of (**a**) −196 °C specimen 3 and (**b**) −196 °C specimen 4, indicating quasi-brittle fracture characteristics.

The fracture surfaces of the room temperature and −196 °C specimens exhibit different features, which could be related to the ductility of the materials (Figure 4). In general, room temperature specimen 1 exhibited ductile fracture features and large shear lips, and the fracture surface was covered by small dimples, as shown in Figure 4a–c. In general, the fracture surface of cryogenic specimens 3 and 4 exhibited smaller shear lips around the edges and a flat fracture surface (see Figure 4d,g). Dimples in the −196 °C specimens 3 and 4 are shown in Figure 4f,i, respectively. Moreover, the −196 °C specimens indicate quasi-brittle fracture characteristics, and both cleavage and ductile fracture features were observed on the fracture surface along with microcracks (Figure 5a,b).

In austenitic stainless steel, a high strain hardening rate enables a high strength level at fracture and retained substantial ductility. However, at −196 °C, deformation occurs due to the rapid transformation of martensite, and the BCC structure is likely to form brittle fractures. Bidulský et al. [14] reported mixed fracture characteristics of an L-PBF-processed and tensile tested 316 L specimen exhibiting cleavage and ductile fracture features at −196 °C. They observed cliffs and quasi cleavage in the ductile region. They also discussed the relationship between the dimple size and mechanical properties, where an increase in dimple size was related to an increase in tensile strength and ductility. Moreover, it was observed that the dimple size increased with decreasing temperature. However, Maicon Rogerio Crivoi et al. [17], Spencer et al. [26], Paredes et al. [42] and Spencer et al. [50], evaluated the fracture surface of 316 L at cryogenic temperatures and suggested ductile fracture since small, shallow dimples were present on the fracture surface. Wenbo et al. [51] discussed the relationship between the average grain size, average dimple size, strength, and ductility for 316 L stainless steels. The grain size and yield strength follow the Hall–Petch relationship. As the grain size increases, the average size of the dimples gradually increases. The large dimples are generally caused by severe plastic deformation, indicating enhanced ductility during the fracture process.

### 3.4. XRD Analysis

**Figure 6:**
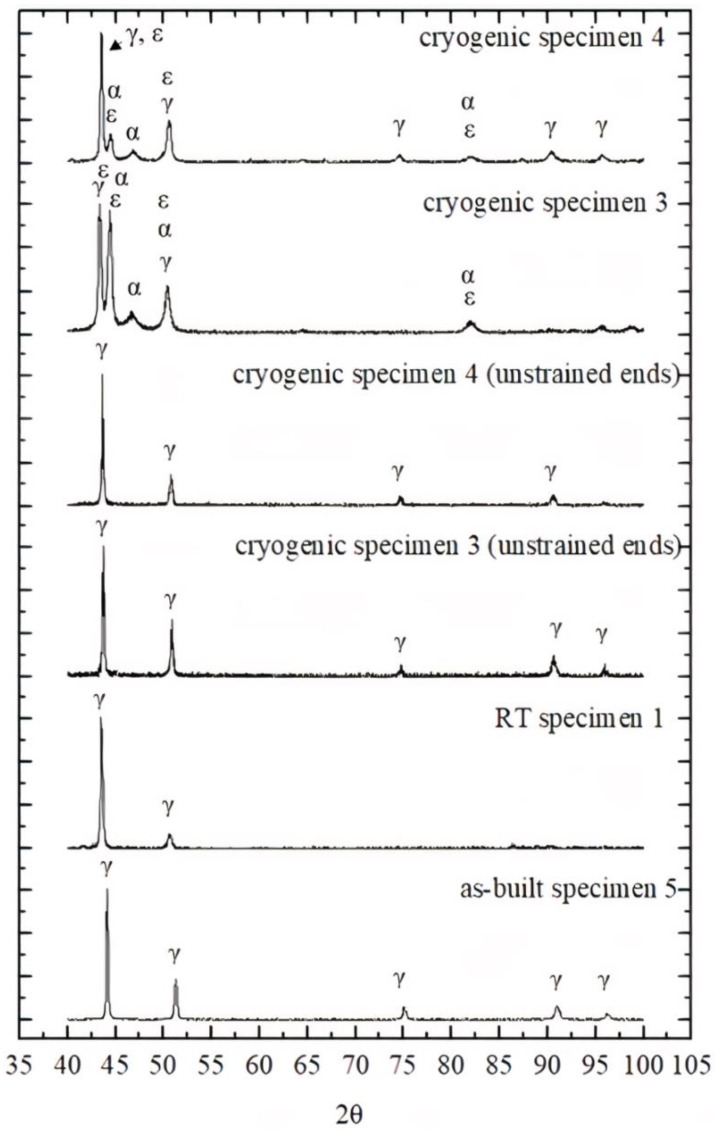
XRD patterns of the room temperature specimen 1, −196 °C temperature specimens 3 and 4, and as-built specimen 5. XRD patterns of the cryogenic specimen 3 and specimen 4 with unstrained ends are also shown.

The XRD patterns of the room temperature specimen 1, −196 °C specimens 3 and 4, and as-built specimen 5 are shown in Figure 6. In addition, the XRD patterns of −196 °C specimens 3 and 4 with unstrained samples from the end of the specimens that were cut into slices for XRD analysis (see Figure 1) are also shown in Figure 6 for comparison.

The XRD patterns of both the as-built specimen 5 and room temperature specimen 1 show peaks attributed solely to the austenite (γ) phase. In the XRD scan, only a single phase of austenite is present after the tensile test at room temperature. Additionally, Liverani et al. [11] and Bartolomeu et al. [52] observed the presence of a singular austenite (γ) phase in as-built specimens. There was no evidence of any other phases.

The XRD patterns of the −196 °C specimens 3 and 4 corresponding to 41% and 16% elongation before fracture were analyzed by Rietveld refinement. The results indicated that specimen 3 consisted of 75% martensite and 25% austenite, while specimen 4 consisted of 50% martensite and 50% austenite.

The XRD patterns of the −196 °C temperature specimens with unstrained ends (specimen 3 and specimen 4) are shown in Figure 6; only austenite peaks are observed, indicating that no martensitic transformation occurred during cooling.

### 3.5. Nanoindentation Tests

**Figure 7:**
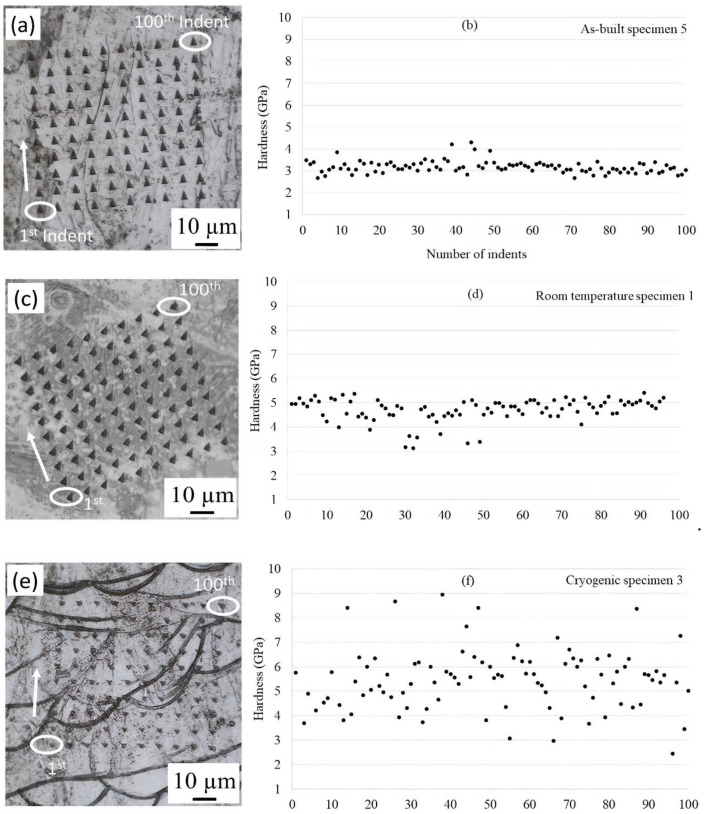

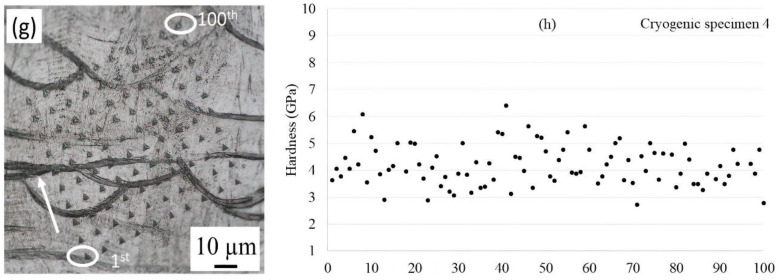
Nanoindentations in 316 L under different conditions: (**a**,**b**) as-built specimen 5, (**c**,**d**) room-temperature specimen 1, (**e**,**f**) −196 °C temperature specimen 3 and (**g**,**h**) −196 °C temperature specimen 4. A load of 60 mN was applied. Indents were performed in a 10 × 10 matrix. The distance between two indents was 12 µm.

The nanoindentation tests indicated that the hardness of the as-built specimen 5, room temperature specimen 1, −196 °C temperature specimen 3 and specimen 4 varied, as shown in Figure 7. Nanoindentation tests were performed on specimens with an etched surface, i.e., different phases appeared on the surface of the specimens. One hundred indents (a 10 × 10 matrix) were performed, with indents transitioning from the left column to the right. The 1st indent, the 100th indent, and the indent direction are shown in Figure 7. In specimen 5, most of the hardness values were approximately 3 GPa, as shown in Figure 7a,b, and there was only one phase of austenite (γ), which was also observed by XRD. In the literature, Roa et al. [53] showed the relationship between the mechanical properties of individual grains and phase transformation mechanisms by using nanoindentation and reported that the nanohardness of γ austenite was 3 GPa.

After tensile testing at room temperature (specimen 1), the nanoindentation hardness values ranged from 3 to 5 GPa, which is higher than that observed for the as-built specimen (Figure 7c,d). Furthermore, the XRD measurements confirmed that no martensitic transformation occurred at room temperature, and thus, no martensite was observed before or after deformation at room temperature. Therefore, the increase in hardness after tensile testing at room temperature could be explained by the deformation and thus the formation of dislocations and twins, as reported by Qiu et al. [34] and Liu et al. [54] for 316 L after tensile testing.

At −196 °C, the nanoindentation result of specimen 3 shows a larger hardness spread in the material, ranging from 3 to 9 GPa (Figure 7e,f). This could be attributed to the new phases formed during tensile testing at −196 °C, as indicated by the XRD results. That is, the softer areas are attributed to austenite, and harder areas are attributed to ε and α martensite. In the −196 °C temperature specimen 4 (Figure 7g,h), the hardness values ranged from 3 to 6 GPa. However, there were very few hardness values above 6 GPa. This indicates that less martensite was present in specimen 4 than in specimen 3, which is in agreement with the Rietveld refinement of the XRD results. Heidarzed et al. [55] reported 6 GPa for the nanohardness of HCP ε martensite in L-PBF samples after high-pressure torsion (HPT). The average Vickers microhardness values of the four specimens are summarized in Table 5. The Vickers microhardness values follow the same trend as the results from the tensile tests. It also agrees with the XRD results and the fraction of microsconstituents, i.e., that the sample containing the largest amount of martensite shows the highest microhardness value and that the as-built material shows the lowest. The increase in plastic deformation and decrease in temperature promote the formation of martensite transformation and increase the hardness of the material. Several parameters influence martensite formation, such as chemical composition, stress applied, plastic strain, and temperature [27,33,42]. The high hardness of martensite results from different origins: the tetragonal lattice contains supersaturated interstitial carbon and a high density of dislocations resulting from the displacive mode of formation [44,56,57,58]. The higher cryogenic lattice resistance initiates thermally activated dislocation slipping. The presence of martensite, which has a very high hardness and strength, and the soft austenite phase in the microstructure contribute to both the high strength and ductility under cryogenic conditions. It is noted that the fine-grained 316 L sample at −196 °C has a higher elastic limit strength than that at RT, and this is attributed to the high cryogenic lattice resistance for initiating thermally activated dislocation slipping as discussed by Jung et al. [19], Li et al. [41], and Sun et al. [59].

## 4. Conclusions

The main objective of this work was to investigate the suitability of L-PBF-built 316 L stainless steel for use in cryogenic applications. The unique microstructure from the L-PBF process was characterized, and mechanical properties were investigated, at room temperature and at −196 °C. The conclusions of this work are as follows:

- There was a significant increase in the yield and ultimate tensile strength at −196 °C compared to room temperature, on average 31% and 86%, respectively. However, the ductility remained at a reasonable level (over 40%) due to the existence of austenite.
- XRD results showed that no martensite formed during cooling to −196 °C or during tensile testing at room temperature. However, both ε and α martensite are detectable in cryogenic samples after tensile testing.
- Different martensite phases were indicated by a variation in the nanohardness values of the cryogenic samples, ranging from 3–9 GPa. The hardness values of the room temperature specimen 1 were in the range of 3–5 GPa.
- The microstructures of the −196 °C temperature specimens were different from that of the room temperature specimen. In addition to the melt pool boundaries, thin, parallel, and plate-like structures were observed, which could indicate martensite inside the grain boundaries in the cryogenic specimen.

## Figures and Tables

**Table 1:** Chemical compositions of 316 L stainless steel powder precursor and 316 L stainless steel bars processed with L-PBF (wt %).

Element	C	Mn	Si	P	S	Cr	Mo	Ni	N	Fe	O
Powder	0.014	1.69	0.70	0.014	0.004	17.8	2.38	12.5	0.09	Bal	165 ppm
L-PBF	0.008	1.43	0.49	0.015	0.007	18.04	2.59	11.77	0.074	Bal	Not measured

**Table 2:** Processing parameters for 316 L stainless steel processed with L-PBF.

Processing Parameters	Contents
Powder size	10–45 μm
Building atmosphere	Argon
Build direction	Horizontal
Scanning speed	900 mm/s
Hatching distance	0.06 mm
Layer thickness	30 μm
Laser power	195 W
Scanning strategy	Meander

**Table 3:** Mechanical properties at room temperature and at −196 °C.

Alloy	Test Temperature	YS (MPa)	UTS (MPa)	Elongation (%)
316 L specimen 1	Room Temperature	570	660	51
316 L specimen 2	Room Temperature	594	689	49
316 L specimen 3	−196 °C	751	1403	41
316 L specimen 4	−196 °C	770	1113	16

**Table 4:** A summary of the yield strength, ultimate tensile strength, and elongation for various 316 alloys conventionally built and L-PBF at room and cryogenic temperature from the literature.

Material	Yield Strength (MPa)	Ultimate Tensile Strength (MPa)	Elongation %	Temperature Condition	Method	References
316 L	554	685	36	RT	L-PBF	[4]
316 L	590	700	36	RT	L-PBF	[6]
316 L	450	640	59	RT	L-PBF	[6]
316 L	499	564	35	RT	L-PBF	[14]
316 L	726	1083	53	77K	L-PBF	[14]
316 L	314	1235	49	77K	Conventional	[15]
316 L	216	529	65	RT	Conventional	[15]
316	590	1196	33	113K	Conventional	[18]
316	494	1328	38	113K	Conventional	[18]
316 L	220	520	40	RT	Conventional	[32]
316 L	270	680	45	RT	Conventional	[32]
316 L	663	685	25	RT	L-PBF	[32]
316 L	602	664	30	RT	L-PBF	[32]
316 L	557	591	42	RT	L-PBF	[32]
316 L	555	684	50.7	RT	L-PBF	[34]
316 L	561	688	50.9	RT	L-PBF	[34]
316 L	487	594	49	RT	L-PBF	[35]
316 L	456	703	45	RT	L-PBF	[36]
316 L	496	717	28	RT	L-PBF	[37]
316 L	345	563	30	RT	Conventional	[37]
316L	310	620	30	RT	Conventional	[38]
316L	468	600	33	RT	Conventional	[39]

**Table 5:** Microhardness of L-PBF 316 L.

Alloy	HV0.1
Specimen 1 (RT)	651 ± 19
Specimen 3 (−196 °C)	1046 ± 87
Specimen 4 (−196 °C)	735 ± 69
Specimen 5 (As-built condition)	439 ± 27

## Data Availability

The data presented in this study are available on request from the corresponding author.
